# Controlled Substance Prescribing Patterns Among Fatal Overdose Decedents with an Opioid, Stimulant, or Both Contributing to Death — Pennsylvania, 2017–2022

**DOI:** 10.15585/mmwr.mm7412a2

**Published:** 2025-04-10

**Authors:** Stephanie Hayden, Stanley M. Murzynski, Ashley Bolton, Carrie Thomas Goetz

**Affiliations:** ^1^Bureau of Epidemiology, Pennsylvania Department of Health; ^2^Office of Drug Surveillance and Misuse Prevention, Pennsylvania Department of Health; ^3^Pennsylvania Office of Administration, Harrisburg, Pennsylvania.

**Summary**

**What is already known about this topic?**

Rates of psychostimulant (stimulant)-related overdose deaths in the United States have increased substantially since 2010; in 2022, 32% of all overdose deaths involved stimulants.

**What is added by this report?**

In Pennsylvania, opioid prescribing (as opposed to stimulant prescribing) during the 3 years preceding death was more common among decedents whose overdose deaths involved stimulants and was more common among all decedents, regardless of the drugs contributing to death.

**What are the implications for public health practice?**

Pre-overdose opioid prescribing is a risk marker for fatal overdose from opioids alone, stimulants alone, or both; pre-overdose stimulant prescribing might not be a risk marker for fatal overdose attributable to stimulants. Identifying risk factors specific to stimulant misuse could better guide development of harm reduction practices to prevent fatal stimulant-related overdoses.

## Abstract

Psychostimulant (stimulant)-related overdose death rates have increased sharply in the United States since 2010, and in 2022, 32% of all U.S. overdose deaths involved stimulants. Data on deaths during 2017–2022 from CDC’s State Unintentional Drug Overdose Reporting System were linked to 2014–2022 Pennsylvania Prescription Drug Monitoring Program data; the Pennsylvania Department of Health’s Office of Drug Surveillance and Misuse Prevention analyzed controlled substance dispensation patterns during the 3 years preceding death among overdose decedents for whom opioids, stimulants, or both contributed to death; statistical analyses were performed on prescription drug dispensation patterns. Comparing overdose deaths in 2022 with those in 2017, deaths involving opioids without stimulants decreased from 2,974 to 1,995, deaths involving stimulants without opioids increased from 300 to 549, and deaths involving both opioids and stimulants increased from 1,703 to 2,346. Irrespective of whether an opioid, stimulant, or both contributed to death, decedents filled more opioid (67.7%, 74.1%, and 63.9%, respectively) than stimulant (10.6%, 11.6%, and 13.4%, respectively) prescriptions preceding death. A higher proportion of stimulant overdose decedents without an opioid contributing to death (74.1%) filled opioid prescriptions compared with decedents whose deaths involved opioids without stimulants or both opioids and stimulants (67.7% and 63.9%, respectively). Opioid prescribing, rather than stimulant prescribing, might be an important potential risk factor for stimulant-related overdose death.

## Introduction

The response to the U.S. drug overdose epidemic has focused on opioid-related overdose deaths. However, during the past decade, the number of psychostimulant (stimulant)-related overdose deaths has increased, with approximately 34,000 people dying from a drug overdose involving stimulants with abuse potential in 2022, accounting for approximately 32% of all drug overdose deaths that year (*1*–*3*). Although an established link between prescription opioid use and opioid-related overdose deaths has been demonstrated (*4*), the relationship between prescription stimulant use and stimulant-related overdose deaths isn’t as clear (*5*,*6*). During 2017–2022, both controlled substance prescribing and drug overdose death trends in Pennsylvania shifted to reflect an increase in stimulant dispensing as well as an increase in stimulant-related overdose deaths, despite a decrease in overall unintentional drug overdose deaths. The evolution of these trends signals the need for a better understanding of potential risk factors contributing to the increase in stimulant-related overdose deaths, such as controlled substance prescribing preceding death. To better understand risk factors for stimulant-related overdose deaths, prescription drug dispensation patterns were analyzed using 2017–2022 overdose death data from CDC’s State Unintentional Drug Overdose Reporting System (SUDORS) and 2014–2022 data from the Pennsylvania Prescription Drug Monitoring Program (PA PDMP).

## Methods

### Data Sources and Classification of Drug Involvement with Death

SUDORS data (*7*) from 2017–2022, which link death certificate data from the Pennsylvania Department of Health’s Bureau of Health Statistics and Registries to toxicology data from participating coroners and medical examiners, were used for this analysis. Data were restricted to three overdose decedent groups characterized by the drug or drugs contributing to death: 1) opioid overdose deaths without a stimulant involved (opioids without stimulants), 2) stimulant overdose deaths without an opioid involved (stimulants without opioids), and 3) overdose deaths involving both opioids and stimulants (opioids and stimulants). For these analyses, stimulants were defined as amphetamine, cocaine, methamphetamine, or other prescription stimulants. Decedent data were linked to 2014–2022 PA PDMP data using Match*Pro probabilistic data linkage software,* linking by patient first name, last name, and date of birth. Decedents’ controlled substance dispensation data were limited to the 3 years of PA PDMP data preceding each decedent’s date of death. Demographic characteristics were tabulated by overdose decedent group and PA PDMP record status.

### Data Analysis

Dispensation patterns were analyzed by performing Pearson’s chi-square tests on the number of decedents by drug schedule,^†^ drug class, final drug class dispensed preceding death, and the number of decedents who filled opioid (opioid-opioid) prescriptions or opioid and benzodiazepine (opioid-benzodiazepine) prescriptions that overlapped by 5 days’ supply or 1 days’ supply, respectively. Kruskal-Wallis tests were performed on the number of dispensations, number of days’ supply, daily morphine milligram equivalents (MMEs)^§^ per decedent, and interval from last dispensation to death. P-values <0.05 were considered statistically significant. All analyses were performed using SAS software (version 9.4; SAS Institute). The Pennsylvania Department of Health’s Institutional Review Board Primary Review Team determined that this study was exempt from the Federal Policy for the Protection of Human Research Subjects.

## Results

### Drug Overdose Deaths, 2017–2022

Among 30,045 drug overdose deaths meeting SUDORS criteria reported during 2017–2022 in Pennsylvania, 28,053 (93%) contained contributing drug information, including 14,315 (51.0%) involving opioids without stimulants, 2,608 (9.3%) involving stimulants without opioids, and 11,130 (39.7%) involving both opioids and stimulants (Table 1). Comparing overdose deaths in 2022 with those in 2017, deaths involving opioids without stimulants decreased from 2,974 to 1,995, deaths involving stimulants without opioids increased from 300 to 549, and deaths involving both opioids and stimulants increased from 1,703 to 2,346. Overdose decedents were predominately male (70.8%), non-Hispanic White persons (73.1%), had a high school education or less (74.5%), and lived in urban-designated areas^¶^ (80.4%). Decedents whose overdoses involved opioids without stimulants or both opioids and stimulants tended to be younger than those involving stimulants without opioids.

**TABLE 1:** Demographic characteristics of overdose decedents, by cause of death drug category and Pennsylvania Prescription Drug Monitoring Program record status (N = 28,053) — Pennsylvania, 2017–2022

Characteristic	Cause of death drug category, no. (%)*								
	At least one PDMP dispensation during the 3 years preceding death								
	Opioids without stimulants			Stimulants without opioids			Opioids and stimulants		
	Yes	No	Total	Yes	No	Total	Yes	No	Total
**Year of death**									
**2017–2022**	**6,869 (48.0)**	**7,446 (52.0)**	**14,315 (100)**	**1,158 (44.6)**	**1,450 (55.4)**	**2,608 (100)**	**6,710 (60.3)**	**4,420 (39.7)**	**11,130 (100)**
2017	805 (27.1)	2,169 (72.9)	**2,974 (20.8)**	80 (26.7)	220 (73.3)	**300 (11.5)**	1,056 (62.0)	647 (38.0)	**1,703 (15.3)**
2018	1,034 (44.3)	1,301 (55.7)	**2,335 (16.3)**	154 (43.4)	201 (56.6)	**355 (13.6)**	900 (64.2)	502 (35.8)	**1,402 (12.6)**
2019	1,071 (49.3)	1,100 (50.7)	**2,171 (15.2)**	197 (46.5)	227 (53.5)	**424 (16.3)**	987 (63.0)	579 (37.0)	**1,566 (14.1)**
2020	1,414 (57.4)	1,048 (42.6)	**2,462 (17.2)**	236 (53.4)	206 (46.6)	**442 (16.9)**	1,247 (63.8)	708 (36.2)	**1,955 (17.6)**
2021	1,393 (48.6)	985 (41.4)	**2,378 (16.6)**	229 (42.6)	309 (57.4)	**538 (20.6)**	1,214 (56.3)	944 (43.7)	**2,158 (19.4)**
2022	1,152 (57.7)	843 (42.3)	**1,995 (13.9)**	262 (47.7)	287 (52.3)	**549 (21.1)**	1,306 (55.7)	1,040 (44.3)	**2,346 (21.1)**
**Sex**									
Female	2,344 (56.5)	1,806 (43.5)	**4,150 (29.0)**	401 (50.3)	396 (49.7)	**797 (30.6)**	2,235 (68.8)	1,013 (31.2)	**3,248 (29.2)**
Male	4,525 (44.5)	5,640 (55.5)	**10,165 (71.0)**	757 (41.8)	1,054 (58.2)	**1,811 (69.4)**	4,475 (56.8)	3,407 (43.2)	**7,882 (70.8)**
**Age group, yrs**									
<25	379 (32.8)	776 (67.2)	**1,155 (8.1)**	13 (25.5)	38 (74.5)	**51 (2.0)**	262 (49.3)	269 (50.7)	**531 (4.8)**
25–34	1,686 (41.1)	2,412 (58.9)	**4,098 (28.6)**	121 (41.6)	170 (58.4)	**291 (11.2)**	1,723 (58.7)	1,211 (41.3)	**2,934 (26.4)**
35–44	1,776 (48.0)	1,925 (52.0)	**3,701 (25.9)**	216 (48.0)	234 (52.0)	**450 (17.3)**	2,018 (61.1)	1,286 (38.9)	**3,304 (29.7)**
45–54	1,414 (52.7)	1,269 (47.3)	**2,683 (18.7)**	331 (45.5)	397 (54.5)	**728 (27.9)**	1,515 (62.2)	920 (37.8)	**2,435 (21.9)**
55–64	1,247 (58.7)	879 (41.3)	**2,126 (14.9)**	396 (45.9)	466 (54.1)	**862 (33.1)**	1,034 (62.3)	625 (37.7)	**1,659 (14.9)**
≥65	367 (66.7)	183 (33.3)	**550 (3.8)**	81 (36.0)	144 (64.0)	**225 (8.6)**	158 (59.4)	108 (40.6)	**266 (2.4)**
**Race and ethnicity** ^†^									
Black or African American, NH	784 (46.9)	887 (53.1)	**1,671 (11.7)**	421 (41.2)	600 (58.8)	**1,021 (39.1)**	1,134 (51.8)	1,057 (48.2)	**2,191 (19.7)**
White, NH	5,665 (50.2)	5,621 (49.8)	**11,286 (78.8)**	684 (48.4)	728 (51.6)	**1,412 (54.1)**	5,144 (65.7)	2,685 (34.3)	**7,829 (70.3)**
Hispanic or Latino	346 (29.6)	822 (70.4)	**1,168 (8.2)**	44 (32.4)	92 (67.6)	**136 (5.2)**	362 (37.9)	592 (62.1)	**954 (8.6)**
Other, NH	74 (38.9)	116 (61.1)	**190 (1.3)**	9 (23.1)	30 (76.9)	**39 (1.5)**	70 (44.9)	86 (55.1)	**156 (1.4)**
**Education**									
High school or less	4,993 (47.5)	5,524 (52.5)	**10,517 (73.5)**	848 (43.6)	1,096 (56.4)	**1,944 (74.5)**	5,042 (59.7)	3,410 (40.3)	**8,452 (75.9)**
Some college	1,307 (49.8)	1,317 (50.2)	**2,624 (18.3)**	182 (47.3)	203 (52.7)	**385 (14.8)**	1,119 (63.1)	655 (36.9)	**1,774 (15.9)**
Bachelor’s degree	329 (46.2)	383 (53.8)	**712 (5.0)**	57 (44.2)	72 (55.8)	**129 (4.9)**	300 (64.0)	169 (36.0)	**469 (4.2)**
Master’s degree or above	102 (59.6)	69 (40.4)	**171 (1.2)**	22 (45.8)	26 (54.2)	**48 (1.8)**	67 (67.7)	32 (32.3)	**99 (0.9)**
Undetermined	138 (47.4)	153 (52.6)	**291 (2.0)**	49 (48.0)	53 (52.0)	**102 (3.9)**	182 (54.2)	154 (45.8)	**336 (3.0)**
**Urbanicity^§^**									
Rural	1,378 (45.5)	1,648 (54.5)	**3,026 (21.1)**	174 (45.3)	210 (54.7)	**384 (14.7)**	1,367 (65.9)	708 (34.1)	**2,075 (18.6)**
Urban	5,491 (48.6)	5,798 (51.4)	**11,289 (78.9)**	984 (44.2)	1,240 (55.8)	**2,224 (85.3)**	5,343 (59.0)	3,712 (41.0)	**9,055 (81.4)**

**Abbreviations**: NH = non-Hispanic; PDMP = prescription drug monitoring program.

* Percentages in total columns are column percentages per characteristic; all other percentages are row percents per characteristic.

^†^ Other, NH race includes all other races not designated as Black or African American, NH or White, NH.

^§^ Counties were designated as rural or urban on the basis of the Health Resources & Services Administration’s classification criteria. https://www.hrsa.gov/rural-health/about-us/what-is-rural

### Controlled Substances Dispensed During the 3 Years Preceding Death

At least one Schedule II–V controlled substance dispensation was recorded in the PA PDMP data during the 3 years preceding death for 6,869 (48.0%) decedents whose overdoses involved opioids without stimulants, 1,158 (44.6%) whose overdoses involved stimulants without opioids, and 6,710 (60.3%) decedents whose overdoses involved both opioids and stimulants (Table 2). A small percentage of decedents with a PA PDMP record filled at least one stimulant prescription during the 3 years preceding death, including 10.6% of those whose deaths involved opioids without stimulants, 11.6% of those whose deaths involved stimulants without opioids, and 13.4% of those whose deaths involved both opioids and stimulants. A higher percentage of decedents whose deaths involved stimulants without opioids (74.1%) filled opioid prescriptions during the 3 years preceding death than decedents whose deaths involved opioids without stimulants or both opioids and stimulants (67.7% and 63.9%, respectively). The percentage of decedents with opioid dispensations with a >5 days’ supply overlap was higher among those whose deaths involved opioids without stimulants (23.0%) than among those whose deaths involved stimulants without opioids (11.1%) or both opioids and stimulants (12.6%) (p<0.001). 

**TABLE 2:** Characteristics of overdose decedents with at least one drug dispensation during the 3 years preceding death, by cause of death drug category — Pennsylvania, 2017–2022

Characteristic*	Cause of death drug category, no. (%)			
	Opioids without stimulants (n = 6,869)	Stimulants without opioids (n = 1,158)	Opioids and stimulants (n = 6,710)	p-value^†^
**Drug schedule^§^**				
II	4,477 (65.2)	805 (69.5)	4,150 (61.8)	<0.001
III	3,142 (45.7)	348 (30.1)	3,412 (50.8)	<0.001
IV	3,835 (55.8)	644 (55.6)	3,309 (49.3)	<0.001
V	652 (9.5)	115 (9.9)	532 (7.9)	0.002
**Drug class dispensed^§^**				
Benzodiazepine	3,011 (43.8)	429 (37)	2,479 (36.9)	<0.001
Buprenorphine	2,501 (36.4)	204 (17.6)	2,856 (42.6)	<0.001
Opioid	4,651 (67.7)	858 (74.1)	4,286 (63.9)	<0.001
Stimulant	728 (10.6)	134 (11.6)	899 (13.4)	<0.001
Other	1,525 (22.2)	239 (20.6)	1,242 (18.5)	<0.001
**Final drug class prescription filled before death**				
Benzodiazepines	1,561 (22.7)	231 (19.9)	1,276 (19.0)	<0.001
Buprenorphine	1,816 (26.4)	147 (12.7)	2,166 (32.3)	<0.001
Opioids	2,775 (40.4)	607 (52.3)	2,477 (36.9)	<0.001
Stimulants	278 (4.0)	75 (6.3)	389 (5.8)	<0.001
Other	439 (6.4)	98 (8.5)	402 (6.0)	0.006
**At least one overlapping prescription**				
Opioid-benzodiazepine^¶^	1,558 (22.7)	191 (16.5)	1,054 (15.7)	<0.001
Opioid-opioid**	1,578 (23.0)	128 (11.1)	847 (12.6)	<0.001

* Characteristics are not mutually exclusive: a single decedent could be counted more than once within each group because of dispensations of various drug schedules, drug classes, or both.

^†^ On the basis of the chi-square test of independence; p-values <0.05 were considered statistically significant.

^§^ The U.S. Drug Enforcement Administration classifies drugs into five distinct categories depending on the drug’s acceptable medical use and abuse potential. Schedule I drugs have no current medically accepted use; Schedule V drugs have the least potential for abuse. https://www.dea.gov/drug-information/drug-scheduling

^¶^ Opioid and benzodiazepine prescriptions for which the number of days’ supply of prescriptions filled overlapped in time by >1 day.

** Opioid prescriptions for which the number of days’ supply of prescriptions filled overlapped in time by >5 days.

In addition, the percentage of decedents with opioid and benzodiazepine dispensations with a >1 days’ supply overlap was higher among those whose deaths involved opioids without stimulants (22.7%) than among those whose deaths involved stimulants without opioids (16.5%) or both opioids and stimulants (15.7%) (p<0.001). Among decedents whose deaths involved either stimulants without opioids or both opioids and stimulants, the median number of any controlled substance dispensations (7.5 and 10.0, respectively) and opioid dispensations (3.0 each), as well as the median MMEs for opioid dispensations (29.6 and 31.2, respectively) were lower than those among decedents whose deaths involved opioids without stimulants (14 controlled substance dispensations, five opioid dispensations, and 33.8 MMEs) (p<0.001) (Table 3). Whereas median days’ supply of stimulants was consistent among all decedent groups (30.0 days), the median days’ supply of opioid dispensations was higher among those whose deaths involved opioids without stimulants (7.0 days) compared with those whose deaths involved stimulants without opioids (5.3 days) or both opioids and stimulants (5.0 days).

**TABLE 3:** Controlled substance prescribing patterns among overdose decedents with at least one dispensation during the 3 years preceding death, by cause of death drug category — Pennsylvania, 2017–2022

Characteristic	Cause of death drug category, median (IQR)			p-value^†^
	Opioids without stimulants	Stimulants without opioids	Opioids and stimulants	
**No. of dispensations**				
Any drug*	14.0 (3.0–41.0)	7.5 (2.0–30.0)	10.0 (3.0–33.0)	<0.001
Opioid	5.0 (2.0–23.0)	3.0 (1.0–10.0)	3.0 (1.0–11.0)	<0.001
Stimulant	10.0 (4.0–23.0)	11.5 (3.0–32.0)	13.0 (4.0–29.0)	0.001
**No. of days’ supply**				
Any drug*	17.2 (7.0–26.2)	15.6 (5.0–29.6)	14.0 (6.1–24.6)	<0.001
Opioid	7.0 (3.5–22.0)	5.3 (3.3–16.5)	5.0 (3.0–15.0)	<0.001
Stimulant	30.0 (29.0–30.0)	30.0 (29.0–30.0)	30.0 (28.8–30.0)	0.20
**Daily morphine milligram equivalent^§^**				
Opioid	33.8 (24.2–55.6)	29.6 (20.4–39.9)	31.2 (22.5–45.0)	<0.001
**No. of days from last dispensation to death**				
Any drug*	68.0 (12.0–335.0)	147.5 (23.0–541.0)	124.5 (17.0–433.0)	<0.001
Opioid	138.0 (16.0–465.0)	299.5 (59.0–669.0)	281.0 (61.0–612.0)	<0.001
Stimulant	50.5 (10.0–301.0)	24.0 (10.0–165.0)	24.0 (10.0–240.0)	0.082

* Any controlled substance recorded in the Pennsylvania Prescription Drug Monitoring Program database.

**^†^** P-values <0.05 were considered statistically significant based on the Kruskal-Wallis test.

^§^ A value that represents the potency of an opioid dose relative to morphine. https://www.cdc.gov/mmwr/volumes/71/rr/rr7103a1.htm

### Interval Between Last Drug Dispensation and Death, by Drug Class

The median number of days between a decedent’s last dispensation and death varied by drug class. Among decedents whose last dispensation preceding death was an opioid, the median interval was shorter among those whose deaths involved opioids without stimulants (138.0 days) than among those whose deaths involved stimulants without opioids and both opioids and stimulants (299.5 and 281.0 days, respectively). However, among decedents whose last dispensation was a stimulant, the median interval was longer among those whose deaths involved opioids without stimulants (50.5 days) than among those whose deaths involved stimulants without opioids or both opioids and stimulants (24.0 days for both groups).

## Discussion

The increase in Pennsylvania overdose deaths involving stimulants without opioids during 2017–2022 permitted exploration of controlled substance prescribing patterns among decedents preceding death. Based on the link between prescription opioid use and risk for opioid overdose death (*4*), it was hypothesized that persons who died from an overdose involving stimulants without opioids might have a history of prescription stimulant dispensations. In this analysis, irrespective of whether an opioid, stimulant, or both contributed to death, only a small percentage of decedents (6.3%) filled a stimulant prescription during the 3 years preceding death, suggesting that receiving a stimulant prescription might not be predictive for subsequent stimulant-involved overdose. In addition, a larger proportion of decedents whose deaths involved stimulants without opioids filled opioid prescriptions during the 3 years preceding death (74%), compared with those whose deaths involved opioids without stimulants (68%) or both opioids and stimulants (64%), suggesting a need for further investigation into the role of opioid prescribing as a potential risk factor for future overdose resulting from the use of nonopioid drugs, including stimulants. In addition, decedents whose last dispensation preceding death was a stimulant had received that prescription closer to their date of death than did decedents whose last dispensation was an opioid. This finding likely relates to the common practice of prescribing and dispensing stimulants monthly over longer periods of time for chronic conditions, such as attention deficit disorder. 

Because of the small proportion of decedents in each group who filled stimulant prescriptions, these findings do not support the hypothesis that increased stimulant prescribing alone is contributing to increases in overdose deaths from stimulants without opioids. Rather, this analysis highlights the implications of opioid prescribing among all overdose decedents, regardless of the drug contributing to death. However, the increasing mortality resulting from stimulant use warrants further analysis, including a longer history of PA PDMP data, enhanced monitoring as new data become available, and investigation of risk factors outside of controlled substance prescribing. In addition, some persons who use opioids have reported that they also use stimulants to compensate for the effects of synthetic opioids (e.g., fentanyl), thereby improving alertness and their ability to function, and this polysubstance use also warrants further exploration (*8*).

### Limitations

The findings in this report are subject to at least three limitations. First, before July 2016, PA PDMP only collected Schedule II drug prescriptions and might not fully characterize controlled substance prescribing patterns among decedents from earlier years of the analysis period (i.e., deaths during January 2017–June 2019). Second, PA PDMP data cannot account for drugs used illicitly by persons for whom they were not prescribed, and many stimulants contributing to overdose and death are used illicitly with few or no approved prescription applications (e.g., cocaine, methamphetamine, and 3,4-methylenedioxymethamphetamine) (*1*). Finally, data from the PA PDMP do not contain information on the condition for which the drug is prescribed and represent controlled substance prescriptions filled, which might not reflect actual use.

### Implications for Public Health Practice

The evolving landscape of the U.S. drug overdose epidemic requires continued evaluation of potential risk factors for overdose. Opioid prescribing should be further investigated as a risk factor for future overdose death resulting from use of nonopioid drugs, such as stimulants; however, the findings in this report highlight the importance of identifying additional overdose risk factors for stimulant-related overdoses. Continued analyses of the latest prescription and overdose death data could identify opportunities for education and intervention if a potential stimulant epidemic emerges.

## Footnotes

*
https://seer.cancer.gov/tools/matchpro/

† The U.S. Drug Enforcement Administration classifies drugs into five distinct categories depending upon the drug’s acceptable medical use and abuse potential. Schedule I drugs have no current medically accepted use; Schedule V drugs have the least potential for abuse. https://www.dea.gov/drug-information/drug-scheduling

§ A value that represents the potency of an opioid dose relative to morphine. https://www.cdc.gov/mmwr/volumes/71/rr/rr7103a1.htm

¶ Counties were designated as rural or urban on the basis of the Health Resources & Services Administration’s classification criteria. https://www.hrsa.gov/rural-health/about-us/what-is-rural
